# l-Galactono-1,4-lactone dehydrogenase is an assembly factor of the membrane arm of mitochondrial complex I in Arabidopsis

**DOI:** 10.1007/s11103-015-0400-4

**Published:** 2015-10-31

**Authors:** Joram Schimmeyer, Ralph Bock, Etienne H. Meyer

**Affiliations:** Max Planck Institute of Molecular Plant Physiology, Am Mühlenberg 1, 14476 Potsdam-Golm, Germany; Institut de Biologie Moléculaire des Plantes du CNRS, 12 rue du général Zimmer, 67084 Strasbourg, France

**Keywords:** Plant mitochondria, Complex I, Complex I assembly, GLDH, Ascorbate biosynthesis, Respiratory chain

## Abstract

l-Galactono-1,4-lactone dehydrogenase (GLDH) catalyses the last enzymatic step of the ascorbate biosynthetic pathway in plants. GLDH is localised to mitochondria and several reports have shown that GLDH is associated with complex I of the respiratory chain. In a *gldh* knock-out mutant, complex I is not detectable, suggesting that GLDH is essential for complex I assembly or stability. GLDH has not been identified as a genuine complex I subunit, instead, it is present in a smaller, lowly abundant version of complex I called complex I*. In addition, GLDH activity has also been detected in smaller protein complexes within mitochondria membranes. Here, we investigated the role of GLDH during complex I assembly. We identified GLDH in complexes co-localising with some complex I assembly intermediates. Using a mutant that accumulates complex I assembly intermediates, we confirmed that GLDH is associated with the complex I assembly intermediates of 400 and 450 kDa. In addition, we detected accumulation of the 200 kDa complex I assembly intermediate in the *gldh* mutant. Taken together, our data suggest that GLDH is an assembly factor of the membrane arm of complex I. This function appears to be independent of the role of GLDH in ascorbate synthesis, as evidenced by the ascorbate-deficient mutant *vtc2*-*1* accumulating wild-type levels of complex I. Therefore, we propose that GLDH is a dual-function protein that has a second, non-enzymatic function in complex I assembly as a plant-specific assembly factor. We propose an updated model for complex I assembly that includes complex I* as an assembly intermediate.

**Electronic supplementary material:**

The online version of this article (doi:10.1007/s11103-015-0400-4) contains supplementary material, which is available to authorized users.

## Introduction

Ascorbate is a natural compound with high antioxidant capacity. Ascorbate represents the predominant form of vitamin C at physiological pH. Some animals, including humans, are unable to synthesise ascorbate and require it as part of their nutrition. Ascorbate can be synthesised through several routes (Smirnoff 2001; Wheeler et al. 2015). In plants, the main biosynthetic pathway involves l-galactose and is also known as the “Smirnoff–Wheeler” pathway (Wheeler et al. 1998). The last step of this pathway is the oxidation of l-galactono-1,4-lactone to l-ascorbate. This step, catalysed by the l-galactono-1,4-lactone dehydrogenase (GLDH) occurs in the mitochondria (Mapson et al. 1954). GLDH transfers electrons to cytochrome *c* (Bartoli et al. 2000; Millar et al. 2003). This suggests that GLDH should be localised in the intermembrane space. However, the exact sub-mitochondrial localization of GLDH remains to be clarified. GLDH has been identified as an integral protein of the inner membrane (Siendones et al. 1999; Bartoli et al. 2000), but it has also been suggested to be a peripheral protein (Leferink et al. 2008).

Because GLDH activity involves electron transfer to cytochrome *c*, ascorbate synthesis is associated with the mitochondrial respiratory chain. In particular, several reports have linked GLDH to complex I (NADH:ubiquinone oxidoreductase). When complex I is engaged, GLDH activity is inhibited by rotenone, an inhibitor of complex I (Millar et al. 2003). A Blue-Native-PAGE analysis of mitochondrial multiprotein complexes identified GLDH in a smaller form of complex I, called complex I* (Heazlewood et al. 2003). Recently, an in-gel activity staining for GLDH has been developed and the activity of GLDH has been detected in three different membrane complexes, including complex I* (Schertl et al. 2012). Surprisingly, GLDH is required for the accumulation of complex I, as complex I is not detectable in the *gldh* mutant (Pineau et al. 2008).

Complex I is the first complex of the mitochondrial electron transfer chain (mETC). It oxidises NADH and transfers electrons onto ubiquinone. Simultaneously, it pumps protons across the mitochondrial inner membrane, contributing to the proton gradient that is used by the ATP synthase to generate ATP (Brandt 2006; Hirst 2013). Complex I is the largest complex of the mETC: It contains more than 40 subunits in eukaryotes and is organised in two arms: a matrix arm that transfers the electrons from NADH to ubiquinone and a membrane arm that is responsible for proton translocation (Sazanov 2014, 2015). In plants, the mature complex I has a size of 1000 kDa. It is made of at least 44 subunits, including 9 proteins expressed from the mitochondrial genome and synthesized in the mitochondria matrix (Meyer 2012; Peters et al. 2013; Braun et al. 2014). In addition, a smaller form of complex I with a size of about 850 kDa, called complex I* has been observed in Blue-Native gels (Heazlewood et al. 2003; Pineau et al. 2008; Meyer et al. 2011; Schertl et al. 2012). Complex I* is able to oxidise NADH in vitro but, based on a genetic analysis, we proposed that complex I* is inactive in vivo (Kühn et al. 2015). When compared to complex I, complex I* contains at least one extra subunit: GLDH (Heazlewood et al. 2003). However, the exact composition of complex I* has not been investigated. Recently, GLDH has been found in two additional mitochondrial complexes of 470 and 420 kDa (Schertl et al. 2012). Using a proteomic approach, a few complex I subunits were found to co-localize with GLDH, however, the presence of GLDH and complex I subunits in the same complexes was not demonstrated (Schertl et al. 2012). GLDH was proposed to play a role during complex I assembly (Schertl et al. 2012) but this role has not yet been firmly demonstrated.

The assembly of complex I has been mainly studied in fungi and mammals (Vogel et al. 2007a). It is a stepwise process during which modules containing a few subunits (assembly intermediates) are assembled together to form the holocomplex. In non-photosynthetic eukaryotes, complex I assembly initiates with the formation of the ubiquinone binding pocket around the subunit Nad1. Then, both arms are elongated by the addition of blocks preassembled in the matrix or in the membrane (Vogel et al. 2007b; McKenzie and Ryan 2010). The last module to be added is the NADH-binding module (Vartak et al. 2014). Several complex I assembly factors have been described in mammals (McKenzie and Ryan 2010; Mimaki et al. 2012; Vartak et al. 2014). Complex I assembly factors are proteins that are essential for complex I accumulation but are not present in the mature complex. Most of the assembly factors have been found to be associated with assembly intermediates (Mimaki et al. 2012). Their specific roles during the assembly process are not clearly understood. In addition, several complex I assembly factors have been shown to be dual-function proteins. For example, ACAD9 is also involved in fatty acid β-oxidation (Haack et al. 2010) and AIF is a mitochondrial flavoprotein playing a role during apoptosis (Vahsen et al. 2004).

In plants, the assembly of complex I is even less well understood. So far, it has only been investigated using a genetic approach. The analysis of Arabidopsis (*Arabidopsis thaliana*) mutants lacking complex I subunits allowed us to propose a model for the assembly pathway of the membrane arm of complex I (Meyer et al. 2011). This model resembles the one described for complex I assembly in mammals, but also shows some plant-specific features. For example, the plant-specific subunit CA2 plays an important role in the early steps of the assembly of the membrane arm. This suggests that specific assembly factors might be required for the assembly of complex I in plants. To date, only one mitochondrial protein has been proposed to play a role during the assembly of complex I, GLDH. Indeed, GLDH is essential for complex I accumulation (Pineau et al. 2008) and is absent from mature complex I (Meyer 2012; Peters et al. 2013). However, the exact role of GLDH during complex I assembly and whether or not this role is linked to GLDH function in ascorbate synthesis, have remained unclear.

Here we have analysed the role of GLDH during complex I assembly in Arabidopsis and have identified GLDH in complex I assembly intermediates. Moreover, we show that in the absence of GLDH, the assembly of complex I arrests at an early stage, confirming a role of GLDH in the assembly of the membrane arm of complex I. Finally, using a genetic approach, we show that the function of GLDH in complex I assembly is independent of its role in ascorbate synthesis. In addition, this work suggests that complex I* is an assembly intermediate of complex I. Based on these findings, we propose an updated model for complex I assembly.

## Material and methods

### Plant material and plant growth

Seeds were surface sterilized with 70 % (v/v) ethanol containing 0.5 % (v/v) Triton 100× and sown in vitro on MS media (0.5× MS, 1 % sucrose, 0.7 % agar). After 2 weeks, seedlings were transferred to soil and grown under a 16 h light (150 µE, 22 °C)/8 h dark (20 °C) photoperiod.

### Mitochondria isolation and fractionation

The aerial organs of 6-week-old plants were harvested and purification of mitochondria was performed according to Meyer et al. (2009). Mitochondrial fractionation, mitoplasts preparation, proteinase K and alkali treatments were performed as described in Spielewoy et al. (2001).

### Preparation of total membranes

Two-week-old seedlings were ground in a mortar in the presence of extraction buffer (0.3 M sucrose, 5 mM tetrasodiumpyrophosphate (10H_2_O), 2 mM EDTA, 10 mM KH_2_PO_4_, 1 % PVP-40, 1 % BSA, 20 mM ascorbic acid, pH 7.5). After filtration through one layer of Miracloth, the filtrate was centrifuged at 2000*g* for 10 min at 4 °C. The supernatant was transferred to a new tube and centrifuged at 35,000*g* for 20 min at 4 °C. The pellet was resuspended in ACA buffer (750 mM aminocaproic acid, 0.5 mM EDTA, 50 mM Bis–Tris-HCl pH 7.0).

### Electrophoresis

Separation of mitochondrial proteins by 12 % SDS-PAGE, separation of complexes by Blue-Native-PAGE (BN-PAGE) and transfer of proteins onto PVDF membranes were performed as described in Meyer et al. (2011). Native molecular weight markers (66–669 kDa, GE Healthcare) were used to estimate the size of complexes and assembly intermediates in BN gels. Gels were stained with colloidal Coomassie G250.

### Western blotting

PVDF membranes were stained, after protein transfer, with a solution of 40 % (v/v) methanol/7 % (v/v) acetic acid containing 0.05 % (w/v) Coomassie Blue R250 for 5 min. The background was reduced by several washing steps using a solution of 40 % (v/v) methanol/7 % (v/v) acetic acid. After scanning using a flatbed scanner, the membranes were fully destained by incubation in 100 % (v/v) methanol. The membranes were then rinsed with water and incubated in blocking buffer [5 % (w/v) milk powder in TBS-Tween20 0.1 % (v/v)] for 1 h at 20 °C. The membranes were then transferred into the primary antibodies solution [2 % (w/v) milk powder in TBS-Tween20 0.1 % (v/v)] and incubated for 16 h at 4 °C. Secondary antibodies linked to horseradish peroxidase were used. The signals were detected by chemiluminescence using the ECL prime kit (GE Healthcare) and a Luminescent Image Analyzer (G-Box-Chemi XT4, Syngene). The following primary antibodies were used: anti-GLDH (AS06182, Agrisera) at a 1:5000 dilution, anti-CAs (Perales et al. 2005) at a 1:10,000 dilution, anti-MnSOD (AS09524, Agrisera) at a 1:5000 dilution, anti-Nad9 (Lamattina et al. 1993) at a 1:50,000 dilution, anti-Cox2 (AS04053A, Agrisera) at a 1:10,000 dilution, anti-Cytc (AS08343A, Agrisera) at a 1:5000 dilution and anti-TOM20-3 (Lister et al. 2007) at a 1:5000 dilution.

## Results

### Topology of GLDH

**Fig. 1:**
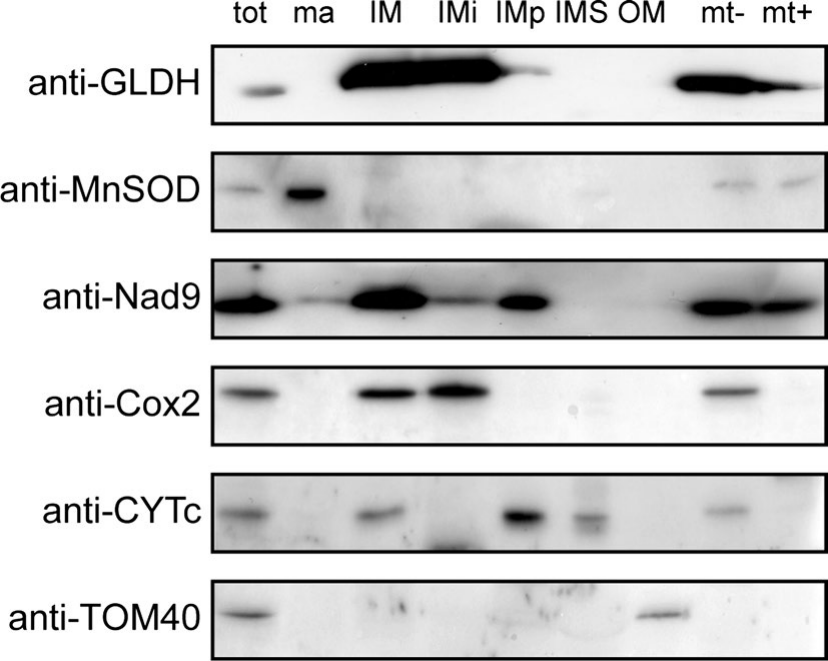
Submitochondrial localization of GLDH. Purified mitochondria (tot) were fractionated and the following submitochondrial compartments were isolated: matrix (ma), inner membrane (IM), intermembrane space (IMS) and outer membrane (OM). Integral proteins of IM (IMi) and peripheral proteins of IM (IMp) were separated by performing an alkali treatment of the IM (see “Material and methods”). In addition, mitochondria were swollen to obtain mitoplasts (swollen mitochondria lacking the outer membrane). Mitoplasts (mt−) were treated with proteinase K to digest the protein domains of the IM facing the IMS (mt+). The different fractions were separated by SDS-PAGE and western blots using anti-GLDH antibodies and antibodies against marker proteins for each compartment were performed

The GLDH is a protein located in (or at) the mitochondrial inner membrane (IM). It catalyses the last step of ascorbate synthesis by oxidising l-galactone-1,4-lactone to ascorbate and transferring two electrons to cytochrome *c* (Bartoli et al. 2000; Millar et al. 2003). This activity suggests that the active site of GLDH is present in the intermembrane space. However, the enzyme has previously been shown to be integral (Siendones et al. 1999) or peripheral (Leferink et al. 2008) to the IM. To clarify the topology of GLDH, we purified mitochondria from wild-type plants and fractionated them to separate the different sub-mitochondrial compartments: the matrix (ma), the IM, the intermembrane space (IMS) and the outer membrane (OM). We also separated integral (IMi) and peripheral (IMp) proteins from the IM fraction using an alkali treatment. In addition, mitochondria were swollen to disrupt the outer membrane and we obtained mitoplasts (mt−). We treated the mitoplasts with proteinase K to digest the protein domains facing the intermembrane space (mt+). We performed control immunodetections against marker proteins to assess the quality of the fractionation (Fig. 1). MnSOD was used as a marker for the matrix (ma). The complex I subunit Nad9 is a peripheral protein of the IM facing the matrix (detected in IM, Imp, mt− and mt+). The complex IV subunit Cox2 is an integral protein of the IM with the recognised epitopes facing the IMS (detected in IM, IMi and mt−). Cytochrome *c* (CYTc) is present in the IMS but was also found to be associated with the IM (detected in IM, IMp, IMS and mt−). Finally, we used TOM20-3 as a marker of the OM. GLDH was detected in the IM fraction and was absent from the matrix, the IMS and the OM. GLDH behaves as an integral protein of the IM facing the IMS, as evidenced by its detection in IMi and mt− and the low signal intensity in mt+ (Fig. 1).

To predict the presence of transmembrane domains, we performed in silico analyses using HMMTOP (Tusnady and Simon 1998) and TMHMM (Krogh et al. 2001). With both algorithms, GLDH is predicted to contain one transmembrane domain located close to the N-terminal end of the protein. However, this transmembrane domain is present in the mitochondrial targeting peptide predicted by Mitoprot (Claros and Vincens 1996). We used the MASC Gator platform (Joshi et al. 2011) to survey the peptides corresponding to GLDH that had been experimentally identified in proteomic analyses in Arabidopsis. Only one out of several hundred peptides was identified in the predicted targeting peptide (Supplementary Fig. 1). The proteomic data suggest that the targeting peptide is cleaved off after import of GLDH into mitochondria and, therefore, the putative transmembrane domain is absent from the mature protein. Together, these analyses indicate that, despite the absence of a discernible transmembrane segment, GLDH behaves as an integral membrane protein of the IM and faces the intermembrane space.

### GLDH is present in complex I assembly intermediates

**Fig. 2:**
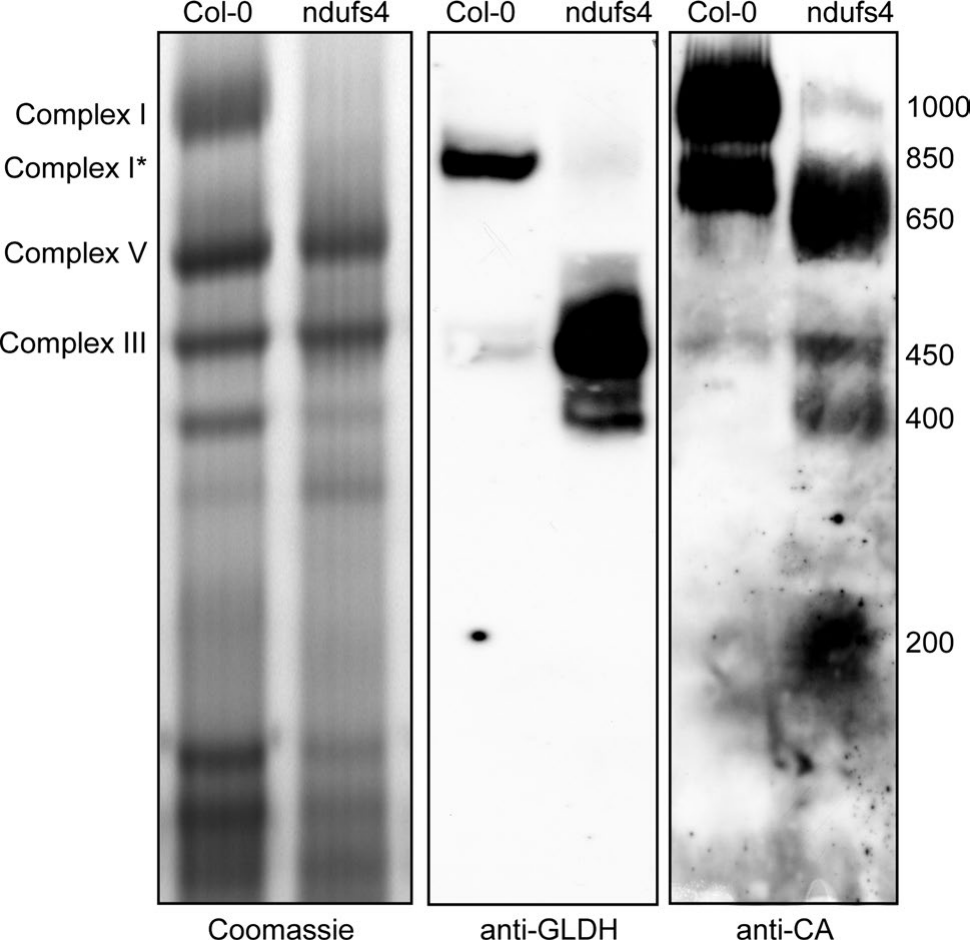
Localisation of GLDH by BN-PAGE. Mitochondrial complexes of Col-0 and the complex I mutant *ndufs4* were resolved by Blue-Native PAGE. Duplicate gels were run and either stained with Coomassie (*left panel*) or transferred onto a membrane for western blot analysis with anti-GLDH (*middle panel*) or anti-CA (*right panel*) antibodies. The position of selected respiratory complexes is indicated on the *left*. The size (in kDa) of complex I and the different assembly intermediate of complex I is indicated on the *right*

**Fig. 3:**
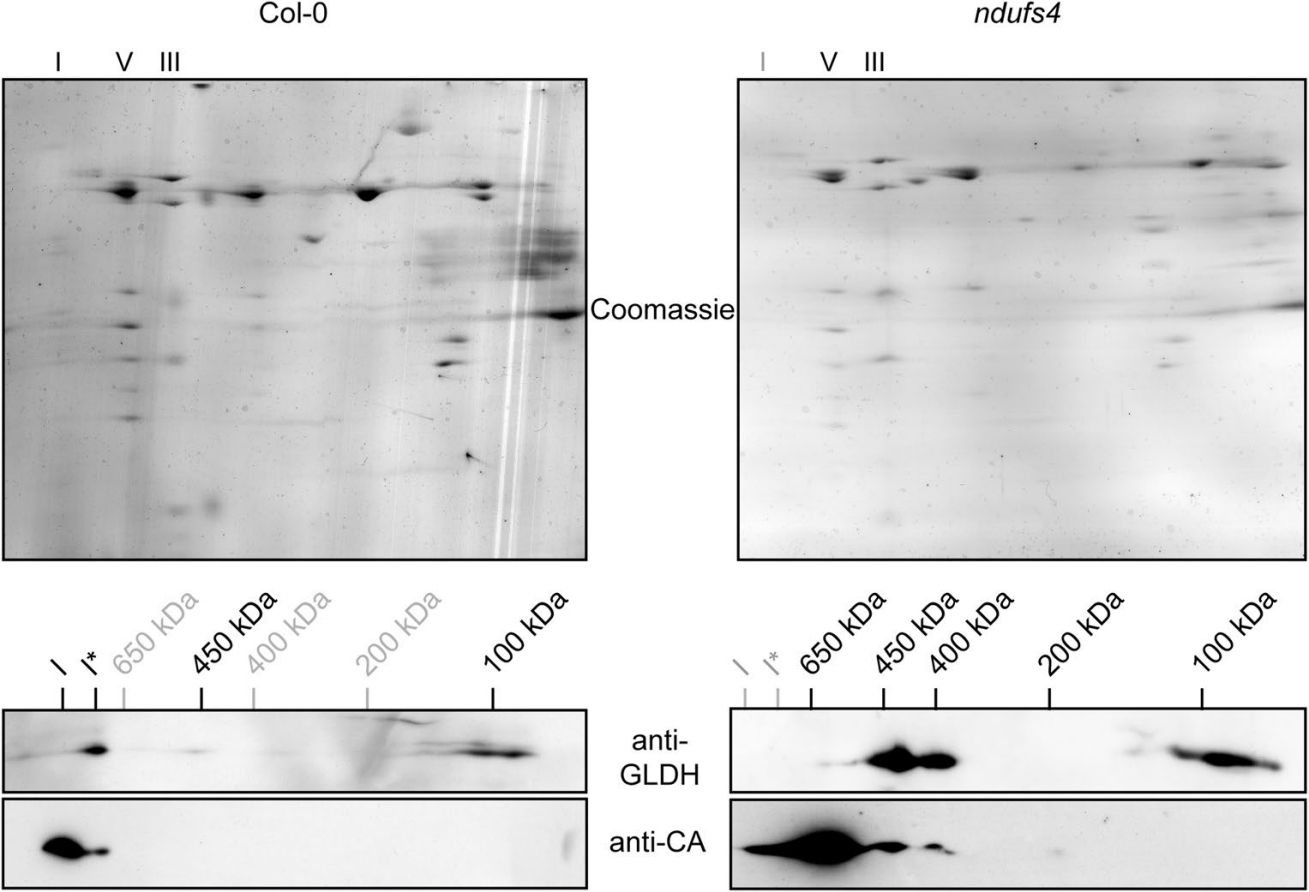
Localisation of GLDH by two-dimensional BN-SDS-PAGE. Mitochondrial complexes of Col-0 and the complex I mutant *ndufs4* were resolved by BN-SDS-PAGE. Duplicate gels were run and either stained with Coomassie (*top panels*) or transferred onto a membrane for western blot analysis with anti-GLDH (*middle panels*) or anti-CA (*bottom panels*) antibodies. The positions of complexes I, III and V of the OXPHOS system are indicated above the *top panel*. The positions of complex I and its assembly intermediates are indicated above of the *central panels*. Complexes not detected in the western blots are indicated in *grey* to facilitate comparison between the two samples

GLDH has been demonstrated to be important for complex I stability (Pineau et al. 2008). It was also shown to localise to three distinct mitochondrial complexes (Schertl et al. 2012). To determine whether or not these three complexes are complex I assembly intermediates, we investigated the localisation of GLDH in the *ndufs4* mutant. NDUFS4 is a subunit localised in the matrix arm of complex I (Vinothkumar et al. 2014). In *ndufs4*, many subunits of the matrix arm of complex I are reduced in abundance (Meyer et al. 2009), and four assembly intermediates of the membrane arm accumulate (Meyer et al. 2011). This suggests that, in *ndufs4*, the matrix arm is not assembled, whereas the membrane arm and its assembly intermediates accumulate. We purified mitochondria from wild type (Col-0) and *ndufs4* plants and separated mitochondrial complexes by Blue-Native PAGE (BN-PAGE). In Col-0, GLDH is detected at the level of complex I* (Fig. 2). A weak signal is also detectable in a complex of 450 kDa. In the *ndufs4* mutant, GLDH is detected in complexes of 450 and 400 kDa (Fig. 2). These GLDH-containing complexes strongly (over)accumulate in *ndufs4* as compared to Col-0. We used the anti-CA antibodies to detect carbonic anhydrase as marker for complex I assembly intermediates. The carbonic anhydrase (CA) subunits of complex I are inserted early in the assembly of the membrane arm of complex I and are therefore present in all the previously identified assembly intermediates (Meyer et al. 2011). Four complex I assembly intermediates were detected in *ndufs4* at 200, 400, 450 and 650 kDa. As the 400 and 450 kDa CA-containing complexes accumulate in the *ndufs4* mutant, our western blot analysis suggests that the complexes containing GLDH in *ndufs4* correspond to complex I assembly intermediates (Fig. 2). We confirmed that the observed signals are specific by separating the subunits of the different complexes by BN-SDS-PAGE. In Col-0, GLDH is found in complex I* (Fig. 3, left panel) and, in *ndufs4*, it is present in the complex I assembly intermediates of 400 and 450 kDa (Fig. 3, right panel). In both genotypes, GLDH is also detected in the lower part of the BN-PAGE (around 100 kDa). Interestingly, whenever GLDH is detected in high molecular weight complexes, it co-localizes with the CA subunits of complex I. Altogether, these analyses indicate that GLDH is present in three complex I-related protein complexes: the assembly intermediates of 400 and 450 kDa and complex I*. However, it is absent from the 200 and 650 kDa assembly intermediates that also overaccumulate in *ndufs4*.

### In the absence of GLDH, complex I assembly stops early

**Fig. 4:**
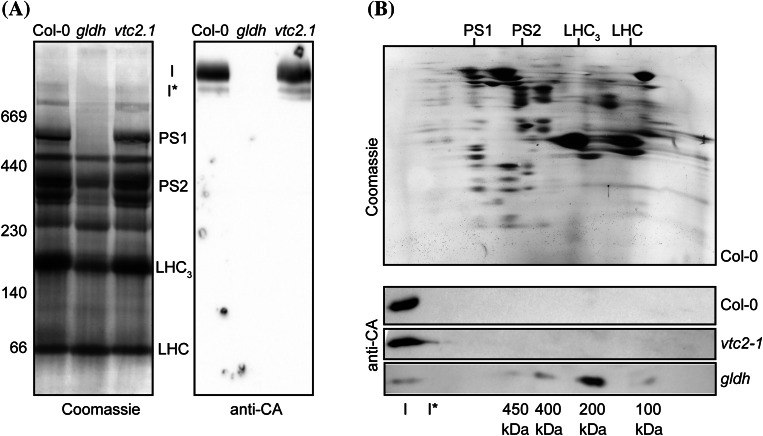
Characterisation of complex I assembly in the *gldh* mutant. Total membranes were purified from Col-0 and the ascorbate-deficient mutants *gldh* and *vtc2*-*1*. **a** BN-PAGE analysis of membrane complexes. The *left panel* shows the Coomassie staining of the gel. The *right panel* shows the western blot analysis using anti-CA antibodies. The sizes of the marker bands of the molecular weight marker used for calibration of the gel are indicated in kDa on the *left*. The positions of complex I (I), complex I* (I*), photosystem 1 (PS1), photosystem 2 (PS2), the LHC trimers (LHC_3_) and LHC monomers (LHC) are indicated between *both panels*. **b** BN-SDS-PAGE analysis of membrane complexes. *Top panel* Coomassie staining of a representative gel (obtained for Col-0). The positions of photosystem 1 (PS1), photosystem 2 (PS2), the LHC trimers (LHC_3_) and LHC monomers (LHC) are indicated on the *top*. The *bottom three panels* show western blot analyses using anti-CA antibodies. The plant line analysed is indicated on the *right*. The positions of complex I (I) and the assembly intermediates detected in the *gldh* mutant are indicated in the *bottom of the panel*

To confirm the function of GLDH as a complex I assembly factor, we investigated the assembly of complex I in the *gldh* mutant. GLDH could play two distinct roles during complex I assembly. First, it could play a structural or stabilizing role for specific assembly intermediates. Second, it could indirectly be essential through providing the ascorbate that might be required during the assembly process. To distinguish between these hypotheses, we included in our analysis the *vtc2*-*1* mutant. This mutant accumulates very low amount of ascorbate due to a mutation in the cytosolic GDP-l-galactose phosphorylase (Conklin et al. 2000; Linster et al. 2007). Because of the severe phenotype of *gldh*, we were unable to extract mitochondria from this mutant. As an alternative, we purified a crude membrane fraction from young seedlings of Col-0, *gldh* and *vtc2*-*1* and performed a BN-PAGE analysis. We solubilized and separated the membrane complexes by BN-SDS-PAGE and analysed complex I composition by western blot using the anti-CA antibodies. We were unable to detect complex I in the *gldh* mutant, but we observed normal amount of complex I in the *vtc2*-*1* mutant (Fig. 4a). When separating the complexes by BN-SDS-PAGE, we found that a 200 kDa complex containing CA accumulates in the *gldh* mutant (Fig. 4b). This complex corresponds to a previously described complex I assembly intermediate (Meyer et al. 2011). This observation confirms that, in the assembly pathway, GLDH is incorporated into the membrane arm of complex I during the transition from the 200 kDa intermediate to the 400 kDa intermediate. We also detected very low amounts of complex I and other assembly intermediates in the *gldh* mutant, suggesting that the assembly of complex I is not completely arrested, but it is severely impaired in the absence of GLDH. The analysis of the *vtc2*-*1* mutant showed that complex I assembly was not affected in this mutant. Therefore, we conclude that the function of GLDH in complex I assembly may not be linked to its role in ascorbate synthesis.

## Discussion

In this study, we have shown that GLDH acts as an assembly factor for complex I of the mitochondrial electron transfer chain. Our results demonstrate that GDLH is associated with several complex I assembly intermediates. GLDH is not required for the early stages of complex I assembly, as revealed by the accumulation of the 200 kDa assembly intermediate in the *gldh* mutant. Thus, GLDH has all characteristics of an assembly factor: it is important for at least one step of the assembly process (transition from the 200 kDa intermediate to the 400 kDa intermediate), it is associated with some assembly intermediates, but it is absent from the mature complex.

### Sub-mitochondrial localisation of GLDH

We identified GLDH to be associated with complex I* and the assembly intermediates of 400 and 450 kDa. GLDH activity was detected in complexes of very similar size (Schertl et al. 2012). The size difference of the complexes observed in the two studies can be explained by variations in molecular mass calibration of the native gels. We also identified two pools of GLDH, one associated with complex I assembly intermediates and another one present in the lower part of the BN-PAGE. The presence of GLDH in the lower part of native gels has previously been observed in BN-SDS-PAGE analysis of mitochondria membranes (Schertl et al. 2012; www.gelmap.de/1226 spot 116 (Klodmann et al. 2011) www.gelmap.de/530 spots 1102, 1157 and 1215). All these spots are located at the vertical of complexes with a molecular weight around 100 kDa. GLDH is a protein of 58 kDa, therefore the lower molecular weight fraction of GLDH either represents a dimer or a small GLDH-containing complex. While our topological analysis (Fig. 1) suggests that GLDH behaves as an integral protein of the IM, we could not predict any clear transmembrane domain (Fig. S1). Consistent with this, when expressed in *Escherichia coli*, Arabidopsis GLDH is found in the soluble fraction (Leferink et al. 2008). Altogether, these observations suggest that GLDH is tightly tethered to the membrane through protein–protein interactions. The identification of GLDH-interacting protein partners will be required to elucidate the mechanism involved in the association of GLDH with the IM.

### Complex I*

**Fig. 5:**
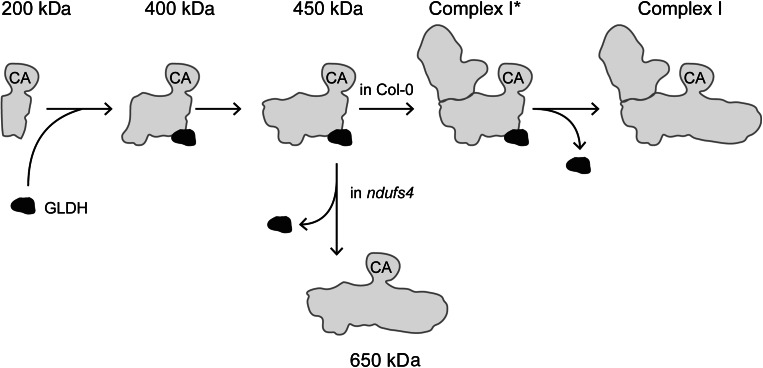
Model showing the different steps of complex I assembly. GLDH is indicated in *black* and complex I assembly intermediates are shown in *grey*. The name of each assembly intermediate is indicated above *each complex* (except for the 650 kDa intermediate). The localisation of the carbonic anhydrases in the complex is indicated by CA. In Col-0, GLDH is incorporated during the transition between the 200 and 400 kDa intermediates, and released when complex I* is converted into complex I. In the *ndufs4* mutant, the matrix arm is not assembled, leading to the full assembly of the membrane arm (intermediate of 650 kDa) after the release of GLDH

In Col-0, GLDH is mainly present in complex I*. In *ndufs4*, complex I* is not assembled and GLDH is not present in any intermediate larger than 450 kDa. This suggests that the 450 kDa intermediate is a precursor of complex I*. We propose that complex I* is an assembly intermediate of complex I. Because complex I* has NADH oxidase activity (Meyer et al. 2011), it should contain the fully assembled matrix arm. According to the published structures of the bacterial and mammalian complex I (Baradaran et al. 2013; Vinothkumar et al. 2014), the matrix arm is anchored at the proximal end of the membrane arm. Therefore, complex I* and its precursor, the 450 kDa intermediate, should contain the proximal part of the membrane arm. Using specific antibodies, Nad6 and CA2 were confirmed to be present in these intermediates (Meyer et al. 2011). In addition, the presence of Nad2, CA2, CA3, GRIM19 and CAL was confirmed by mass spectrometry (Schertl et al. 2012). These subunits are located in the middle part of the membrane arm (Sunderhaus et al. 2006; Efremov and Sazanov 2011). The distal part of the membrane arm contains at least Nad4 and Nad5 (Efremov and Sazanov 2011). In plants, mutants impaired in Nad4 or Nad5 accumulate complex I* (Karpova and Newton 1999; Pineau et al. 2008; Haili et al. 2013; Colas des Francs-Small et al. 2014). Therefore, we conclude that complex I* and the 450 kDa intermediate lack the distal part of the membrane arm. We propose an updated version of the assembly model for complex I that includes complex I* as an assembly intermediate and GLDH as an assembly factor (Fig. 5).

### Role of GLDH during complex I assembly

Our data demonstrate that GLDH is an assembly factor for the membrane arm of complex I. Several assembly factors for complex I have been identified in human mitochondria (McKenzie and Ryan 2010; Mimaki et al. 2012; Vartak et al. 2014). However, since GLDH is not conserved in humans, it represents the first plant-specific assembly factor for complex I. Interestingly, ECSIT, an assembly factor in human mitochondria, interacts with several assembly intermediates of the membrane arm of complex I. It enters the pathway during the formation of the intermediate of 460 kDa and dissociates when the mature complex is formed (Vogel et al. 2007c). Although the exact role of ECSIT during the assembly is unknown, it was proposed to act as stabilizing factor for the assembly intermediates (Vogel et al. 2007c). This proposed function is similar to the one we suggest here for GLDH in Arabidopsis. In addition, as for ECSIT, GLDH is essential for complex I assembly. No genes encoding proteins homologous to ECSIT could be identified in the Arabidopsis genome. GLDH and ECSIT might thus fulfil analogous roles during the assembly of complex I. We propose that GLDH acts as stabilizing factor for complex I intermediates. Elucidation of the exact composition and the structure of the GLDH-containing assembly intermediates will help clarifying the precise function(s) of GLDH in these complexes during complex I assembly.

### Complex I and ascorbate

Our results suggest that GLDH plays two distinct roles in plant mitochondria. GLDH is essential for ascorbate biosynthesis (Mapson et al. 1954; Leferink et al. 2008) and we show here that it is also essential for complex I assembly. Using the *vtc2*-*1* mutant, we uncoupled these two functions. *vtc2*-*1* is an ascorbate deficient mutant that accumulates about 10 % of wild-type levels of ascorbate (Conklin et al. 2000). We found that the *vtc2.1* mutant accumulates normal levels of complex I. This suggests that either ascorbate is not required for the assembly of complex I or low levels of ascorbate are sufficient for complex I assembly. Therefore, we cannot entirely rule out that ascorbate is required for complex I assembly. Alternative routes for ascorbate synthesis may also be present in plants (Lorence et al. 2004). However, these routes are bypassing GLDH and should therefore not be affected in the *gldh* mutant. Thus, if alternative ascorbate synthesis routes exist, *gldh* should also accumulate low levels of ascorbate. Taken together with our analysis of complex I assembly in ascorbate-deficient mutants, this suggests that the function of GLDH during complex I assembly is not linked to its role in ascorbate biosynthesis. We also observed that a fraction of GLDH is not associated with complex I intermediates. Whether this free pool of GLDH is involved in the ascorbate synthesis function or, alternatively, both pools represent enzymatically active GLDH, remains to be investigated.

GLDH catalyses the last step of ascorbate synthesis. A functional link between ascorbate synthesis and respiration has previously been reported (Bartoli et al. 2000; Millar et al. 2003). GLDH uses oxidised cytochrome *c* as electron acceptor. Surprisingly, the electron flow through complex I affects GLDH activity. When complex I is engaged, rotenone inhibits both complex I and ascorbate synthesis (Millar et al. 2003). However, GLDH is not associated with complex I but with complex I* (Fig. 2; Heazlewood et al. 2003). Based on a genetic analysis, we previously suggested that complex I* is not active in vivo (Kühn et al. 2015) and, therefore, the rotenone treatment may only have a structural effect on complex I*. If ascorbate synthesis is inhibited by this putative conformational change in complex I*, it would suggest that the GLDH found in the complexes detected at around 100 kDa is inactive. To date, there is no evidence supporting this hypothesis. Also, rotenone treatment should theoretically favour ascorbate synthesis as the cytochrome *c* pool should be more oxidised. In our view, the inhibition of ascorbate synthesis by rotenone can only be explained if, upon complex I inhibition, oxidised cytochrome *c* becomes unavailable to GLDH. However, this has not yet been experimentally demonstrated. Clearly, the elucidation of the mechanism underlying the regulation of ascorbate synthesis by respiration requires further investigation.

## Electronic supplementary material

Supplementary material 1 (PPTX 359 kb)

## References

[CR1] Baradaran R, Berrisford JM, Minhas GS, Sazanov LA (2013). Crystal structure of the entire respiratory complex I. Nature.

[CR2] Bartoli CG, Pastori GM, Foyer CH (2000). Ascorbate biosynthesis in mitochondria is linked to the electron transport chain between complexes III and IV. Plant Physiol.

[CR3] Brandt U (2006). Energy converting NADH:quinone oxidoreductase (complex I). Annu Rev Biochem.

[CR4] Braun HP, Binder S, Brennicke A, Eubel H, Fernie AR, Finkemeier I, Klodmann J, König AC, Kühn K, Meyer E, Obata T, Schwarzländer M, Takenaka M, Zehrmann A (2014) The life of plant mitochondrial complex I. Mitochondrion. doi:10.1016/j.mito.2014.02.006 10.1016/j.mito.2014.02.006 24561573

[CR5] Claros MG, Vincens P (1996). Computational method to predict mitochondrially imported proteins and their targeting sequences. Eur J Biochem FEBS.

[CR6] Colas des Francs-Small C, Falcon de Longevialle A, Li Y, Lowe E, Tanz SK, Smith C, Bevan MW, Small I (2014). The pentatricopeptide repeat proteins TANG2 and ORGANELLE TRANSCRIPT PROCESSING439 are involved in the splicing of the multipartite nad5 transcript encoding a subunit of mitochondrial complex I. Plant Physiol.

[CR7] Conklin PL, Saracco SA, Norris SR, Last RL (2000). Identification of ascorbic acid-deficient *Arabidopsis thaliana* mutants. Genetics.

[CR8] Efremov RG, Sazanov LA (2011). Structure of the membrane domain of respiratory complex I. Nature.

[CR9] Haack TB, Danhauser K, Haberberger B, Hoser J, Strecker V, Boehm D, Uziel G, Lamantea E, Invernizzi F, Poulton J, Rolinski B, Iuso A, Biskup S, Schmidt T, Mewes HW, Wittig I, Meitinger T, Zeviani M, Prokisch H (2010). Exome sequencing identifies ACAD9 mutations as a cause of complex I deficiency. Nat Genet.

[CR10] Haili N, Arnal N, Quadrado M, Amiar S, Tcherkez G, Dahan J, Briozzo P, Colas des Francs-Small C, Vrielynck N, Mireau H (2013). The pentatricopeptide repeat MTSF1 protein stabilizes the nad4 mRNA in Arabidopsis mitochondria. Nucleic Acids Res.

[CR11] Heazlewood JL, Howell KA, Millar AH (2003). Mitochondrial complex I from Arabidopsis and rice: orthologs of mammalian and fungal components coupled with plant-specific subunits. Biochim Biophys Acta.

[CR12] Hirst J (2013). Mitochondrial complex I. Annu Rev Biochem.

[CR13] Joshi HJ, Hirsch-Hoffmann M, Baerenfaller K, Gruissem W, Baginsky S, Schmidt R, Schulze WX, Sun Q, van Wijk KJ, Egelhofer V, Wienkoop S, Weckwerth W, Bruley C, Rolland N, Toyoda T, Nakagami H, Jones AM, Briggs SP, Castleden I, Tanz SK, Millar AH, Heazlewood JL (2011). MASCP Gator: an aggregation portal for the visualization of Arabidopsis proteomics data. Plant Physiol.

[CR14] Karpova OV, Newton KJ (1999). A partially assembled complex I in NAD4-deficient mitochondria of maize. Plant J.

[CR15] Klodmann J, Senkler M, Rode C, Braun HP (2011). Defining the protein complex proteome of plant mitochondria. Plant Physiol.

[CR16] Krogh A, Larsson B, von Heijne G, Sonnhammer EL (2001). Predicting transmembrane protein topology with a hidden Markov model: application to complete genomes. J Mol Biol.

[CR17] Kühn K, Obata T, Feher K, Bock R, Fernie AR, Meyer EH (2015) Complete mitochondrial complex I deficiency induces an upregulation of respiratory fluxes that is abolished by traces of functional complex I. Plant Physiol 168:1537–1549 10.1104/pp.15.00589 PMC4528760 26134164

[CR18] Lamattina L, Gonzalez D, Gualberto J, Grienenberger JM (1993). Higher plant mitochondria encode an homologue of the nuclear-encoded 30-kDa subunit of bovine mitochondrial complex I. Eur J Biochem FEBS.

[CR19] Leferink NG, van den Berg WA, van Berkel WJ (2008). l-Galactono-γ-lactone dehydrogenase from *Arabidopsis thaliana*, a flavoprotein involved in vitamin C biosynthesis. FEBS J.

[CR20] Linster CL, Gomez TA, Christensen KC, Adler LN, Young BD, Brenner C, Clarke SG (2007). Arabidopsis VTC2 encodes a GDP-l-galactose phosphorylase, the last unknown enzyme in the Smirnoff–Wheeler pathway to ascorbic acid in plants. J Biol Chem.

[CR21] Lister R, Carrie C, Duncan O, Ho LH, Howell KA, Murcha MW, Whelan J (2007). Functional definition of outer membrane proteins involved in preprotein import into mitochondria. Plant Cell.

[CR22] Lorence A, Chevone BI, Mendes P, Nessler CL (2004). myo-inositol oxygenase offers a possible entry point into plant ascorbate biosynthesis. Plant Physiol.

[CR23] Mapson LW, Isherwood FA, Chen YT (1954). Biological synthesis of l-ascorbic acid: the conversion of l-galactono-γ-lactone into l-ascorbic acid by plant mitochondria. Biochem J.

[CR24] McKenzie M, Ryan MT (2010). Assembly factors of human mitochondrial complex I and their defects in disease. IUBMB Life.

[CR25] Meyer EH (2012). Proteomic investigations of complex I composition: how to define a subunit?. Front Plant Sci.

[CR26] Meyer EH, Tomaz T, Carroll AJ, Estavillo G, Delannoy E, Tanz SK, Small ID, Pogson BJ, Millar AH (2009). Remodeled respiration in ndufs4 with low phosphorylation efficiency suppresses Arabidopsis germination and growth and alters control of metabolism at night. Plant Physiol.

[CR27] Meyer EH, Solheim C, Tanz SK, Bonnard G, Millar AH (2011). Insights into the composition and assembly of the membrane arm of plant complex I through analysis of subcomplexes in Arabidopsis mutant lines. J Biol Chem.

[CR28] Millar AH, Mittova V, Kiddle G, Heazlewood JL, Bartoli CG, Theodoulou FL, Foyer CH (2003). Control of ascorbate synthesis by respiration and its implications for stress responses. Plant Physiol.

[CR29] Mimaki M, Wang X, McKenzie M, Thorburn DR, Ryan MT (2012). Understanding mitochondrial complex I assembly in health and disease. Biochim Biophys Acta.

[CR30] Perales M, Eubel H, Heinemeyer J, Colaneri A, Zabaleta E, Braun HP (2005). Disruption of a nuclear gene encoding a mitochondrial gamma carbonic anhydrase reduces complex I and supercomplex I + III2 levels and alters mitochondrial physiology in Arabidopsis. J Mol Biol.

[CR31] Peters K, Belt K, Braun HP (2013). 3D gel map of arabidopsis complex I. Front Plant Sci.

[CR32] Pineau B, Layoune O, Danon A, De Paepe R (2008). l-Galactono-1,4-lactone dehydrogenase is required for the accumulation of plant respiratory complex I. J Biol Chem.

[CR33] Sazanov LA (2014). The mechanism of coupling between electron transfer and proton translocation in respiratory complex I. J Bioenerg Biomembr.

[CR34] Sazanov LA (2015). A giant molecular proton pump: structure and mechanism of respiratory complex I. Nat Rev Mol Cell Biol.

[CR35] Schertl P, Sunderhaus S, Klodmann J, Grozeff GE, Bartoli CG, Braun HP (2012). l-Galactono-1,4-lactone dehydrogenase (GLDH) forms part of three subcomplexes of mitochondrial complex I in *Arabidopsis thaliana*. J Biol Chem.

[CR36] Siendones E, Gonzalez-Reyes JA, Santos-Ocana C, Navas P, Córdoba F (1999). Biosynthesis of ascorbic acid in kidney bean. l-Galactono-γ-lactone dehydrogenase is an intrinsic protein located at the mitochondrial inner membrane. Plant Physiol.

[CR37] Smirnoff N (2001). l-Ascorbic acid biosynthesis. Vitam Horm.

[CR38] Spielewoy N, Schulz H, Grienenberger JM, Thony-Meyer L, Bonnard G (2001). CCME, a nuclear-encoded heme-binding protein involved in cytochrome c maturation in plant mitochondria. J Biol Chem.

[CR39] Sunderhaus S, Dudkina NV, Jansch L, Klodmann J, Heinemeyer J, Perales M, Zabaleta E, Boekema EJ, Braun HP (2006). Carbonic anhydrase subunits form a matrix-exposed domain attached to the membrane arm of mitochondrial complex I in plants. J Biol Chem.

[CR40] Tusnady GE, Simon I (1998). Principles governing amino acid composition of integral membrane proteins: application to topology prediction. J Mol Biol.

[CR41] Vahsen N, Cande C, Briere JJ, Benit P, Joza N, Larochette N, Mastroberardino PG, Pequignot MO, Casares N, Lazar V, Feraud O, Debili N, Wissing S, Engelhardt S, Madeo F, Piacentini M, Penninger JM, Schagger H, Rustin P, Kroemer G (2004). AIF deficiency compromises oxidative phosphorylation. EMBO J.

[CR42] Vartak RS, Semwal MK, Bai Y (2014). An update on complex I assembly: the assembly of players. J Bioenerg Biomembr.

[CR43] Vinothkumar KR, Zhu J, Hirst J (2014). Architecture of mammalian respiratory complex I. Nature.

[CR44] Vogel RO, Smeitink JA, Nijtmans LG (2007). Human mitochondrial complex I assembly: a dynamic and versatile process. Biochim Biophys Acta.

[CR45] Vogel RO, Dieteren CE, van den Heuvel LP, Willems PH, Smeitink JA, Koopman WJ, Nijtmans LG (2007). Identification of mitochondrial complex I assembly intermediates by tracing tagged NDUFS3 demonstrates the entry point of mitochondrial subunits. J Biol Chem.

[CR46] Vogel RO, Janssen RJ, van den Brand MA, Dieteren CE, Verkaart S, Koopman WJ, Willems PH, Pluk W, van den Heuvel LP, Smeitink JA, Nijtmans LG (2007). Cytosolic signaling protein Ecsit also localizes to mitochondria where it interacts with chaperone NDUFAF1 and functions in complex I assembly. Genes Dev.

[CR47] Wheeler GL, Jones MA, Smirnoff N (1998). The biosynthetic pathway of vitamin C in higher plants. Nature.

[CR48] Wheeler G, Ishikawa T, Pornsaksit V, Smirnoff N (2015) Evolution of alternative biosynthetic pathways for vitamin C following plastid acquisition in photosynthetic eukaryotes. eLife. doi:10.7554/eLife.06369 10.7554/eLife.06369 PMC4396506 25768426

