# Supplementary material for: l-Galactono-1,4-lactone dehydrogenase is an assembly factor of the membrane arm of mitochondrial complex I in Arabidopsis
Source: Plant Mol Biol. 2015 Oct 31;90:117–26. doi: 10.1007/s11103-015-0400-4 (PMC4689740; doi:10.1007/s11103-015-0400-4)
Supplement: Supplementary file 1 — Supplementary material 1 (PPTX 359 kb) [file 11103_2015_400_MOESM1_ESM.pptx]

## Slide 1
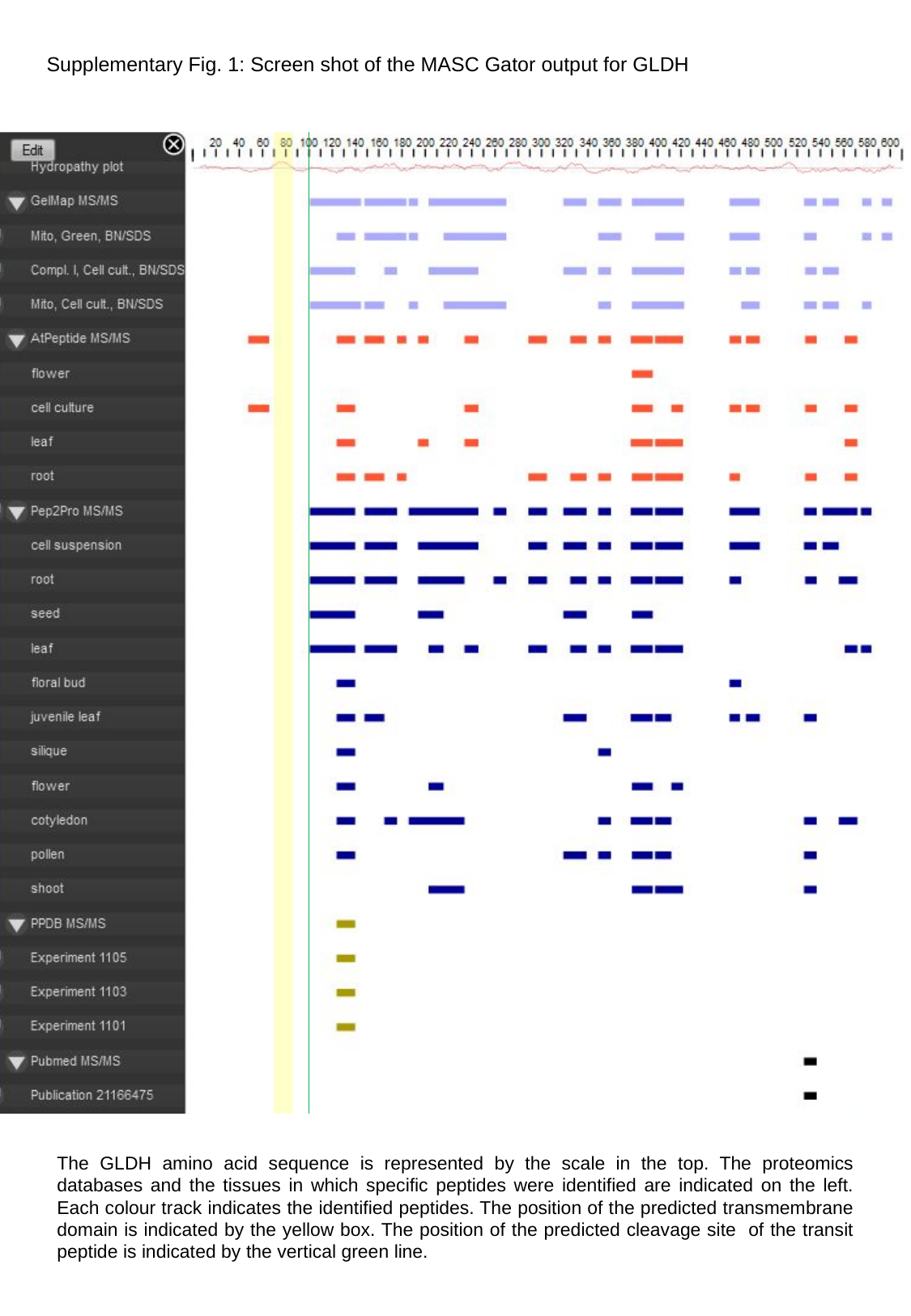

Supplementary Fig. 1: Screen shot of the MASC Gator output for GLDH
The GLDH amino acid sequence is represented by the scale in the top. The proteomics databases and the tissues in which specific peptides were identified are indicated on the left. Each colour track indicates the identified peptides. The position of the predicted transmembrane domain is indicated by the yellow box. The position of the predicted cleavage site of the transit peptide is indicated by the vertical green line.
